# Idiopathic Choroidal Neovascularization in Pregnancy: A Case Report

**DOI:** 10.7759/cureus.34611

**Published:** 2023-02-03

**Authors:** Mohammad Daniyal Monis, Sanja M Ali, Israr A Bhutto, Pir S Mahar

**Affiliations:** 1 Ophthalmology, Al Ibrahim Eye Hospital / Isra Postgraduate Institute of Ophthalmology, Karachi, PAK; 2 Ophthalmology and Visual Sciences, Aga Khan University Hospital, Karachi, PAK

**Keywords:** literature review, pregnant, anti-vegf, intravitreal injections, anti-vascular endothelial growth factor, idiopathic choroidal neovascularization

## Abstract

Choroidal neovascularization (CNV) is the abnormal growth of vessels from the choroidal vasculature to the neurosensory retina through the Bruch's membrane and is usually associated with "wet" age-related macular degeneration (AMD). Other causes include myopia, traumatic rupture of the choroid, multifocal choroiditis, and histoplasmosis. CNV is a major cause of visual loss and treatment is aimed at halting progression and stabilizing vision. Intravitreal anti-vascular endothelial growth factor (IVT anti-VEGF) injection is the treatment of choice for CNV regardless of etiology. However, its use in pregnancy is debatable, due to its mechanism of action and lack of evidence of safety in pregnancy.

Herein, we report a 27-year-old pregnant female, who complained of blurred and decreased vision in her left eye for two weeks. On examination, her unaided vision was 6/6 in her right eye and 6/18 partial in her left eye with no further improvement. Based on history, examination, and investigations she was diagnosed as a case of idiopathic CNV in pregnancy, being only the sixth reported case worldwide. Citing the risk of possible fetal adverse effects, the patient did not consent to the treatment despite extensive counseling. She was advised to follow up regularly and to receive IVT anti-VEGF injections immediately after delivery.

A literature review was therefore undertaken to broaden our understanding of the treatment protocols and outcomes of using IVT anti-VEGF in pregnancy. This helped us to develop an understanding of the possible relative safety of such a treatment when individually tailored with a multi-disciplinary approach.

## Introduction

Choroidal neovascularization (CNV) is described as the “growth of new blood vessels that originate from the choroid through a break in the Bruch’s membrane, into the sub-retinal pigment epithelium/subretinal space”. It is usually associated with age-related macular degeneration (AMD). Myopia, traumatic rupture of the choroid, multifocal choroiditis, and histoplasmosis are among other common causes. CNV can complicate any pathological process and when no causative pathology can be identified, as in our case, the condition is labeled as idiopathic-CNV [1].

In the above-mentioned conditions, marked visual deterioration due to CNV occurs because of distortion of the overlying retina with the release of sub-retinal and intra-retinal fluid. This can be due to various molecular abnormalities, among which the excessive release of vascular endothelial growth factor (VEGF) is known to be the major culprit. Though marked elevation of VEGF is a normal physiological change during pregnancy, it can promote or facilitate the development of CNV by altering the balance between pro- and anti-angiogenic factors [2].

Irrespective of the etiology of CNV, the mainstay of treatment is intravitreal (IVT) anti-VEGF injection. However, due to its mechanism of action, possible embryotoxic and fetal adverse effects, and lack of evidence of its safety in pregnancy, the use of anti-VEGF treatment for CNV in pregnancy is debatable [3-5].

## Case presentation

A 27-year-old pregnant housewife presented to the medical retina clinic at Al-Ibrahim Eye Hospital, Karachi with complaints of sudden blurring and rapidly progressive, painless decrease in near and far visual acuity in her left eye for 15 days. She was in her usual state of health two weeks previously when she noticed the symptoms. She denied seeing flashes, floaters, falling curtain effects, or distortion of images or shapes of objects. She also denied diplopia, and headache and had no known comorbidities, history of ocular trauma, or spectacle use. She was in the second month of her second pregnancy and was taking multivitamins as advised. Her previous pregnancy was uneventful, with no visual complaints, and had a normal vaginal delivery. Her past medical, surgical, and family history was unremarkable.

**Figure 1:**
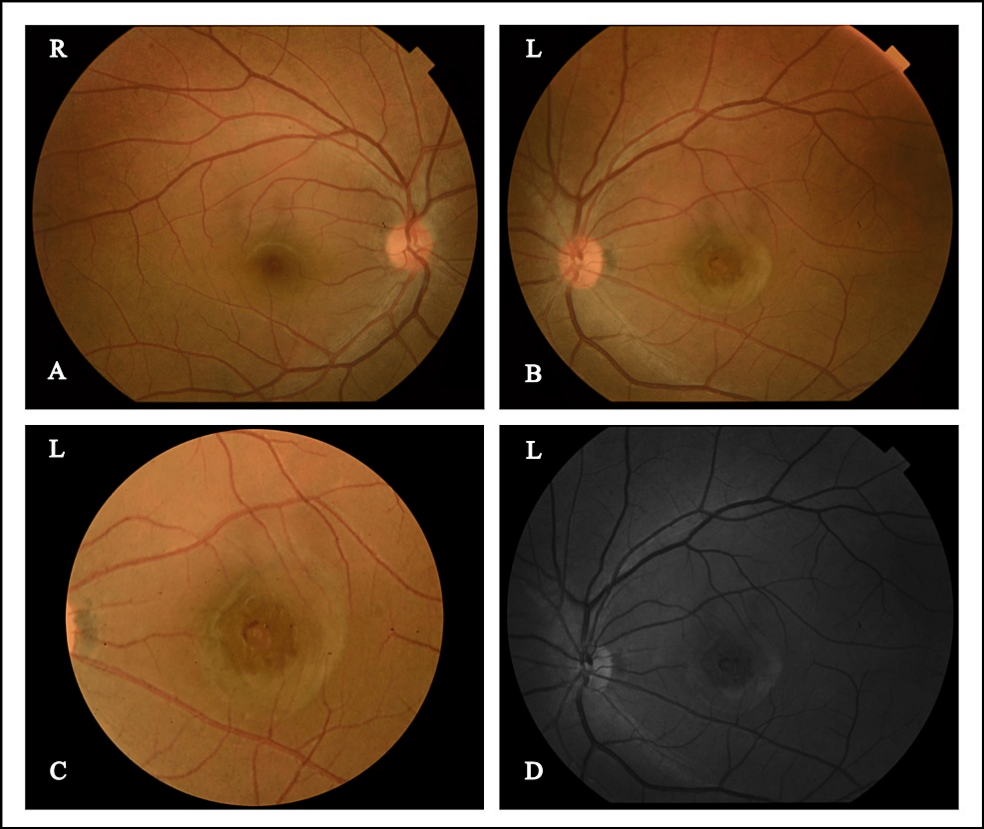
(A,B) Fundus photographs of both eyes; (C) Macula left eye; (D) Red-free photograph of left eye

On examination, her best corrected visual acuity was 6/6 in her right eye and 6/18 in her left eye with a refractive error of -1.25DS / -0.25DC x160o in her right eye and -1.5DS in her left eye. Hirshberg's reflex was central with normal extraocular movements. Slit lamp examination of anterior segments was unremarkable with clear media and 12 mmHg intraocular pressure in her both eyes with Goldmann applanation tonometer. The dilated fundus examination of the right eye was normal with a 0.2 cup-disc ratio as well as a normal macula with a good macular reflex and normal vasculature. However, the posterior segment of the left eye revealed a dull macular reflex with a slightly raised, juxta-foveal, greyish-white lesion measuring about one disc diameter and located two disc diameters temporal to the optic disc margin. No visible blood vessels were seen crossing the lesion and the surrounding retinal vasculature and optic disc were normal (Figure 1).

**Figure 2:**
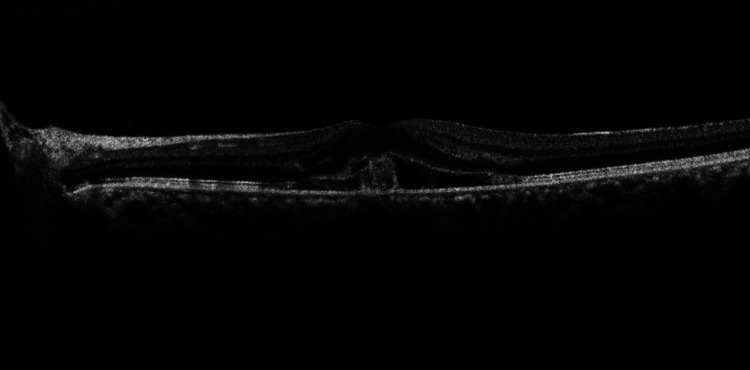
SD-OCT of macula of left eye SD-OCT macula of left eye showing visible disruption of the Bruch’s membrane and retinal pigment epithelium (RPE). Subretinal fluid and hyperreflective material are seen along with distortion of the photoreceptor layer and the ellipsoid zone.

The patient informed seeing distorted paracentral vertical lines, without any breaks on the Amsler grid with her left eye. The Spectral-Domain optical coherence tomography (OCT) and OCT-angiography (OCT RS 3000 Advance, Nidek Japan) of the posterior segment revealed normal anatomy of the right eye. In her left eye, there was visible disruption of Bruch’s membrane and retinal pigment epithelium with a hyperreflective mass arising from the choroid, projecting towards the inner retinal layers resulting in distortion of the photoreceptor layer and ellipsoid zone along with subretinal fluid (Figure 2).

**Figure 3:**
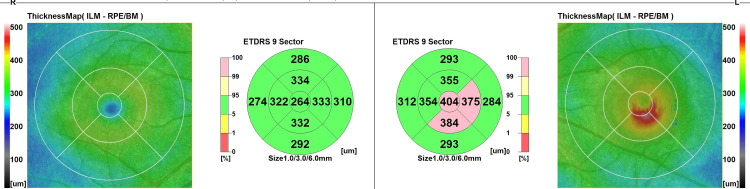
SD-OCT macular map of both eyes showing macular thickness on the EDTRS chart SD- OCT: Spectral domain optical coherence tomography

The OCT macular map quantifies the macular thickening on the EDTRS Chart (Figure 3).

**Figure 4:**
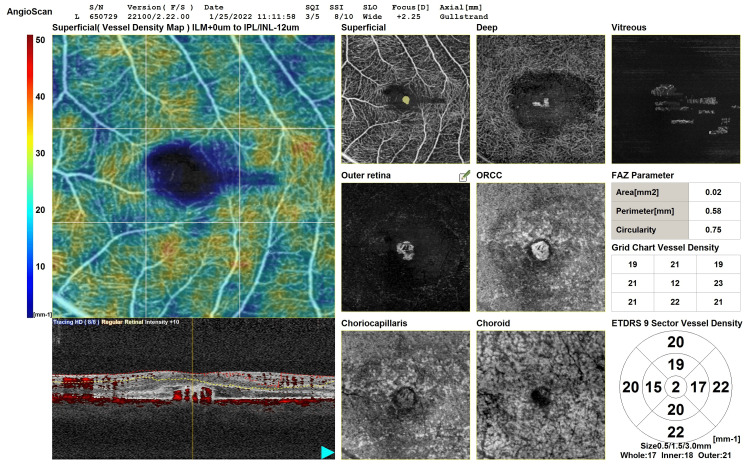
SD-OCTA of left eye SD-OCTA: Spectral domain optical coherence tomography angiography

The OCT-angiography shows the lesion arising from the choriocapillaris, extending to the superficial plexus with considerable foveal avascular zone shrinkage. No other inflammatory findings were noted on OCT-angiography (Figure 4).

Based on the history, clinical findings, and investigations, a diagnosis of idiopathic CNV in pregnancy was made. The patient was informed in detail and counseled about treatment options of anti-VEGF injections given IVT. The risks of unknown complications of this kind of therapy affecting her fetus were also discussed with the patient. After a lengthy discussion, the patient refused anti-VEGF treatment citing the risk of fetal adverse effects. Unfortunately, as photodynamic therapy (PDT) with intravenous Visudyne dye is not currently available in our country, this option was not discussed. The patient was advised for regular follow-ups and IVT anti-VEGF treatment soon after delivery. The patient was unfortunately lost to follow-up and she did not report back for any treatment.

## Discussion

Abnormal choroidal vessels grow through a break in the Bruch’s membrane, into the sub-retinal pigment epithelium and the subretinal space. Along with associated subretinal fluid accumulation, CNV causes disruption and disorganization of the photoreceptors and the inner retinal layers and leads to visual deterioration. It is mostly associated with AMD with about 10% of the patients developing neovascularization, i.e., wet AMD of which 79-90% of untreated patients become legally blind [6,7]. It also occurs secondary to histoplasmosis, multifocal choroiditis, myopia, and traumatic rupture of the choroid. In settings where no primary pathology can be identified, the condition is labeled as idiopathic CNV which is a rare occurrence. It is extremely rare for idiopathic CNV to occur in a previously normal pregnant female, which could be facilitated by the high levels of circulating VEGF, a state normal and essential for pregnancy [1].

Ancillary testing including fundus fluorescein angiography, OCT, and OCTA are used to aid in the diagnosis, quantify and classify the lesion, and evaluate the response to therapy. IVT anti-VEGF injection is the treatment of choice for CNV. However, due to a lack of significant data regarding its safety in pregnancy and due to possible embryotoxic and fetal adverse effects, the use of anti-VEGF in pregnancy is a looming question for ophthalmologists [5]. Other proven treatment options of variable effectiveness include laser photocoagulation, photodynamic therapy with intravenous Visudyne dye injection, and IVT corticosteroid injection for inflammatory pathology [7].

Bamdad et al. used a pregnant rat model for assessing the teratogenic effects of IVT bevacizumab, an anti-VEGF, injection. His experimental work showed decreased fetal weight and teratogenic effects of using IVT bevacizumab in early rat pregnancies while being safe in late pregnancy [8]. With only a few uncontrolled published series and reports on IVT anti-VEGF therapy in human pregnancy and because of the exclusion of pregnant participants from clinical trials, a clear consensus cannot be reached on the matter.

A study by Ben Ghezala et al. is the largest summation of such case reports from France. They reported 228 IVT anti-VEGF or corticosteroid (CS) injections in 139 patients either during pregnancy or the month preceding pregnancy: 93 women received anti-VEGF alone, 39 received CS alone, and seven received both. Of the 153 identified IVT anti-VEGF injections 84.3% of them were administered in either the month preceding pregnancy or in the first trimester, 47(30.7%) and 82 (53.6%) respectively. Among the 94 women studied for comparative analysis, spontaneous or medical termination of pregnancy was reported among 10 (16.1%) women receiving anti-VEGF vs. one woman (3.1%) receiving CS. Fetal lesions or fetal distress was reported in 11 (17.7%) cases receiving anti-VEGF agents vs. four (12.5%) receiving CS. Multivariant analysis showed that anti-VEGF agents were not associated with a higher risk of obstetric and neonatal complications when compared with CS [9].

De Groot et al. reported 14 IVT anti-VEGF injections in six pregnancies for multifocal choroiditis. Elevated IOP was reported in one patient who received concurrent IVT and systemic CS while another patient who developed multiple complications, already had a complicated obstetric history [10].

Throughout the world, miscarriage has been reported as a major pregnancy-associated complication of IVT anti-VEGF, usually occurring soon after injection [11-13]. Contradictory to this proposed association is the prevalence of an already high miscarriage rate (30-40%) among otherwise healthy females. Others have reported uneventful pregnancies with no developmental defects after IVT anti-VEGF administration in pregnancy, though long-term follow-up data is currently not available [4,9]. Administration of IVT anti-VEGF during the embryogenic phase in an otherwise undiagnosed pregnancy has also shown variable results [14,15].

## Conclusions

As more and more data in the form of case reports and series emerges, the confidence in treatment with the anti-VEGF regime in pregnancy is increasing. It should be noted, however, that based on these individual reports, the safety of the said regimen cannot be ascertained as statistically significant data is lacking for absolute certainty. Randomized control clinical studies are needed to assess the optimal therapeutic options for these patients

At-risk patients should be identified through screening programs. We advocate pre-pregnancy counseling to encourage patients to seek treatment before pregnancy, as pregnancy may exacerbate the condition. The decision to treat should be made with caution and only after discussing the benefits and risks with the patient as well as the treating obstetrician, preferably avoiding the first trimester.
